# Practical Medicine Series Year Book

**Published:** 1904-11

**Authors:** 


PRACTICAL MEDICINE SERIES YEAR BOOK. Publishers, 40 Dearborn Street, Chicago.

Therapeutics, Preventive Medicine, Climatology, Forensic Medicine are the departments considered in the issue of this valued series for July, 1904. Advances in materia medica and therapeutics are collected by Dr. Geo. F. Butler and Geo. S. Browning. The noteworthy additions in this province have received attention in a manner sufficiently full for the reader to avail himself of them practically. Preventive medicine is under the care of Dr. Henry B. Fairle, and contains abstracts of the recent literature on the subject of the infectious diseases and sanitation, general and personal. Climatology is edited by Drs. Norman Brige and Edith Claypole, and is a general review of the subject.

